# Anti-Psoriasis Effect of Diclofenac and Celecoxib Using the Tail Model for Psoriasis

**DOI:** 10.3390/pharmaceutics14040885

**Published:** 2022-04-18

**Authors:** Diana Ana-Maria Nițescu, Horia Păunescu, Alina Elena Ștefan, Laurențiu Coman, Corneliu Cristian Georgescu, Andrei Constantin Stoian, Daniela Gologan, Ion Fulga, Oana Andreia Coman

**Affiliations:** 1 Department of Pharmacology and Pharmacotherapy, Faculty of Medicine, “Carol Davila” University of Medicine and Pharmacy, 020021 Bucharest, Romania; diana.nitescu@drd.umfcd.ro (D.A.-M.N.); andrei.stoian@umfcd.ro (A.C.S.); ion.fulga@umfcd.ro (I.F.); oana.coman@umfcd.ro (O.A.C.); 2 Department of Pathology, Faculty of Veterinary Medicine, University of Agronomical Sciences and Veterinary Medicine, 011464 Bucharest, Romania; alina@lumea.net; 3 Department of Physiology, Faculty of Pharmacy, “Carol Davila” University of Medicine and Pharmacy, 020021 Bucharest, Romania; laurentiu.coman@umfcd.ro; 4 Department of Pharmacology and Pharmacotherapy, Faculty of Dental Medicine, Craiova University of Medicine and Pharmacy, 200349 Craiova, Romania; cristian.georgescu@umfcv.ro; 5 Department of Organic Chemistry, Faculty of Applied Chemistry and Materials Science, Polytechnic University of Bucharest, 060042 Bucharest, Romania; daniela.gologan@lumea.net

**Keywords:** psoriasis, topical treatment, orthokeratosis, diclofenac, celecoxib, tretinoin

## Abstract

Non-steroidal anti-inflammatory drugs (NSAIDs) showed effects in some hyperproliferative dermatologic pathologies. The aim of the study is the assessment of anti-psoriasis effect of diclofenac and celecoxib using a mice tail model. The topical application of substances on the proximal mice tails was performed for two weeks. The effects on the epidermal granular layer and mean epidermal thickness (excluding the stratum corneum) were evaluated using hematoxylin–eosin staining. Orthokeratosis degree and percentual drug activity were calculated. A positive control group treated with tretinoin and two negative controls (white soft paraffin and untreated mice) were used. Orthokeratosis degree significantly increased in all the NSAIDs groups (celecoxib 1%, 2% and diclofenac 1%, 2%) and in the tretinoin 0.05% group, versus negative controls. Celecoxib 1% and 2%, tretinoin 0.05% and white soft paraffin significantly increased mean epidermal thickness, versus untreated mice. The values obtained in the case of celecoxib 2% ointment regarding the orthokeratosis degree and percentual drug activity are providing premises for further investigations regarding this effect and the mechanisms of action involved. Celecoxib 2% had the greatest percentual drug activity and is a promising substance for the anti-psoriasis topical treatment. Along with the COX-2 inhibition, celecoxib might have an anti-psoriasis effect by other independent mechanisms.

## 1. Introduction

Psoriasis represents a frequent disease in dermatologic clinical practice. Current treatment is based on traditional systemic therapies and the use of biologic drugs targeting tumor necrosis factor-alpha and interleukins 17 and 23 [1,2,3]. The topical treatment is limited to few substances (dermatocorticosteroids, topical retinoids, calcineurin inhibitors, vitamin D analogs, salicylic acid, anthralin) [4]. Animal research studies ease the development of new anti-psoriasis substances or the formulation improvement of already approved drugs [5]. Psoriasis is characterized by a diminished or even absent granular layer as a result of aberrant keratinocyte differentiation, and this fact can be considered the disease hallmark [6,7].

The non-steroidal anti-inflammatory drugs (NSAIDs) are recently used in dermatological disease treatment, as they inhibit cyclooxygenase (COX) -2 enzyme pathways, that act by stimulating aberrant keratinocyte proliferation [8,9,10]. Recent studies showed that diclofenac is involved in cellular apoptosis regulation of dysplastic keratinocytes [11].

In dermatological pathologies, topical diclofenac is only approved in the treatment of actinic keratosis. There are no clinical trials regarding the anti-psoriasis effect of topically applied diclofenac or celecoxib as resulting from the clinicaltrials.gov database (last accessed on 1 April 2022) and clinicaltrialsregister.eu (last accessed on 1 April 2022). The mechanism of action involved in the treatment of actinic keratoses is by COX-2 inhibition. COX-2 acts by stimulating aberrant keratinocyte proliferation and angiogenesis.

Half-maximal inhibitory concentration (IC50) is a measure of a substance potency in expressing a specific biochemical reaction. IC50 is smaller for diclofenac in comparison with celecoxib, being almost a hundredfold greater for COX-1 and only tenfold smaller for COX-2. Diclofenac is a potent inhibitor of COX-1 and COX-2 but is less selective than celecoxib [12].

At therapeutic concentrations, celecoxib inhibits minimally COX-1, having a COX-2 inhibition intensity almost 30 times higher than of COX-1 [13,14].

The aim of our study was to assess the anti-psoriasis effect of diclofenac and celecoxib, using a mice tail model for psoriasis. We decided to evaluate the effect of two different NSAIDs to see whether a greater COX-2 inhibition would imply a greater in vivo effect on epidermal granular layer development.

The tail model for psoriasis was first developed in 1964 by Jarett and Spearman and was modified later by some authors [15]. This morphometric, sensible, and reproducible model consists of quantitative evaluation of mean epidermal thickness and granular layer development in specimens obtained from mouse tail sections. Two derived parameters, orthokeratosis degree and drug activity percentage, are then calculated.

Retinoids’ effect on keratinocyte differentiation and proliferation are well demonstrated, and we chose tretinoin as a positive control group in order to compare the effect of two NSAIDs (diclofenac, celecoxib) with a known anti-proliferative substance.

## 2. Materials and Methods

### 2.1. Animals

Male Albino mice weighting 20–30 g were used. Animals were provided by the Cantacuzino National Institute of Medico-Military Research and Development. The mice were brought in 4 days before the experiment started for accommodation and were individually housed with ad libitum access to water and food for all the duration of the experiment. The environmental conditions were constant (light, temperature, humidity).

### 2.2. Substances

The substances used (tretinoin, white soft paraffin, diclofenac sodium, celecoxib) are of pharmacopeial quality and were provided from a pure substance supplier (Fagron, Bucharest). Diclofenac sodium, tretinoin and celecoxib were mixed with the white soft paraffin base. White soft paraffin is a semisolid ointment base, easy to apply, that makes an occlusion film that permits a better absorption of active substances when used as a base.

### 2.3. Experimental Design

#### 2.3.1. Animal Groups

The experiment was developed for 14 days as follows.

The animals were divided into 7 groups:

Group 1: negative control (untreated mice)
Group 2: negative control (white soft paraffin)
Group 3: positive control (tretinoin 0.05%)
Group 4: test 1—diclofenac 1%
Group 5: test 2—diclofenac 2%
Group 6: test 3—celecoxib 1%
Group 7: test 4—celecoxib 2%

Two negative control groups were used to evaluate the baseline morphometric aspects of tail tissue. One negative control was of untreated mice and another negative control was with mice treated with white soft paraffin alone. For positive control, a retinoid was used (tretinoin 0.05% in white soft paraffin).

#### 2.3.2. Experimental Protocol

The mice were treated with 0.1 milliliters of ointment left for two hours in the proximal part of the tail, with the aid of a plastic cylinder fixed with an adhesive tape in direct contact with the tail. The quantity of ointment was standardized with the aid of a syringe. At the end of the two hours, the cylinders were removed, and the tails were washed with warm water. This procedure was repeated once a day, 5 days per week, for 2 consecutive weeks. The mice were weighted at 2 days, and the weight curve was similar between groups. At the end of the experiment, the mice were sacrificed after general anesthesia according to the ethical guidelines for lab animals research, and the tails were fixed in 10% neutral-buffered formalin. After dehydration in graded ethanol, clarification in butanol and infiltration with paraffin, sections were cut at 4 µm and stained with Mayer hematoxylin and eosin.

#### 2.3.3. Morphometric Assessment

The evaluation of hematoxylin eosin-stained section was realized using a Zeiss optical microscope. The images evaluation was made with ZEN Blue software.

**Figure 1:**
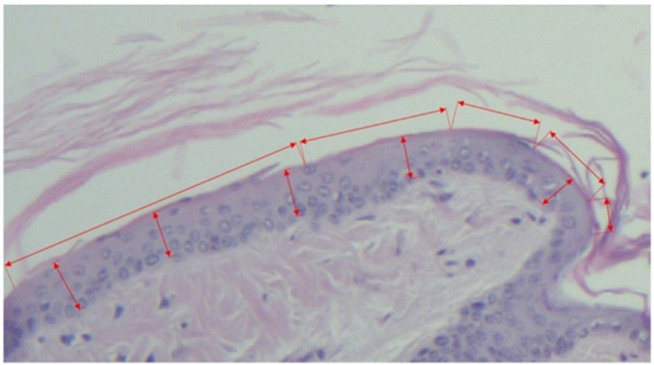
Negative control group section (untreated mice), hematoxylin–eosin staining, 10× objective.

**Figure 2:**
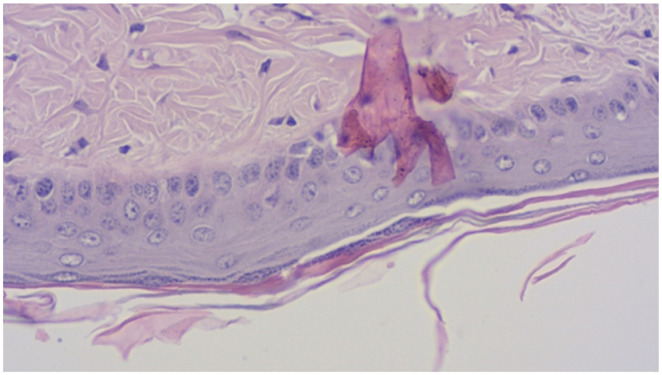
Section with granular layer, hematoxylin–eosin staining, 40× objective.

**Figure 3:**
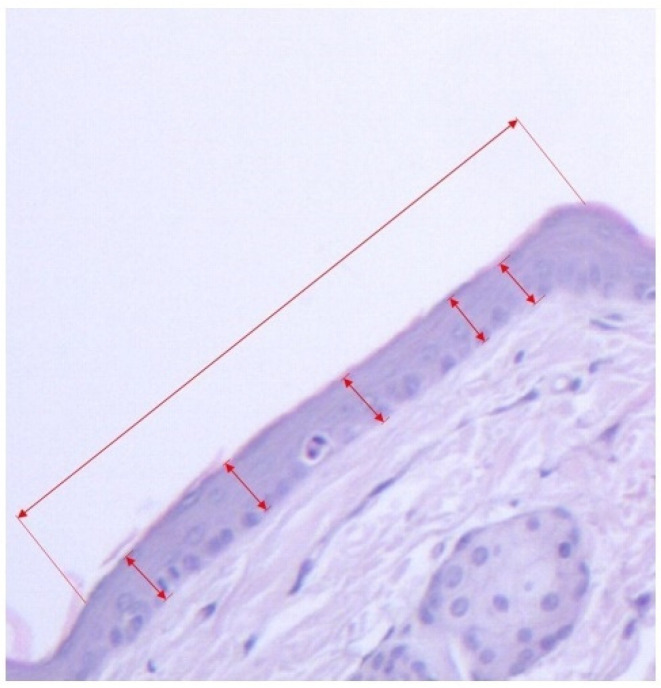
Section group treated with 0.1 milliliter celecoxib 2% applied in the proximal part of the tail. The granular layer extends on the whole length scale, hematoxylin–eosin staining, 10× objective.

The specimens obtained from the experiment were evaluated for:

A. Horizontal length of the continuous granular layer integrated in the scale (see Figure 1, Figure 2 and Figure 3).
B. Horizontal length of the scale, measured as the distance between two follicles including the sebaceous gland (see Figure 1 and Figure 3).
  From the values obtained at A-B the secondary parameters were obtained [4]:
C. The degree of orthokeratosis of the individual scale, measured as the percentual value of A divided by B. For orthokeratosis degree determination, 10 horizontal scales per animal were measured, 60 scales per group. The dimensions were calculated in micrometers.
D. The percentual drug activity was measured as (Oks − Okc)/(100 − Okc) × 100
  Oks = orthokeratosis as mean of the values obtained at D for the test substance (s)
  and
  Okc = orthokeratosis as mean of the values obtained at D for the negative control group 2 (c)—white soft paraffin
E. The vertical length of the epidermic thickness measured between the dermo-epidermic junction and the inferior part of the stratum corneum (see Figure 1 and Figure 3).
F. Mean epidermal thickness of the individual scale (between two pilous follicles). For mean epidermal thickness, 5 measurements were made in each of the 10 scales, resulting in 300 measurements per group. The dimensions were calculated in micrometers.

### 2.4. Statistical Analysis

The statistical analysis was made with Microsoft Excel and SPSS version 25. For statistical comparisons, the Kruskal–Wallis non-parametric test was used, with a level of significance set at *p* ≤ 0.05.

## 3. Results

The values regarding the orthokeratosis degree, mean epidermic thickness and percentual drug activity are presented in Table 1.

**Figure 4:**
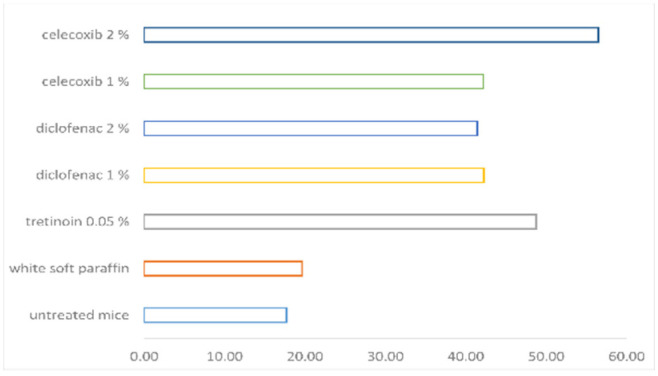
The orthokeratosis degree.

**Figure 5:**
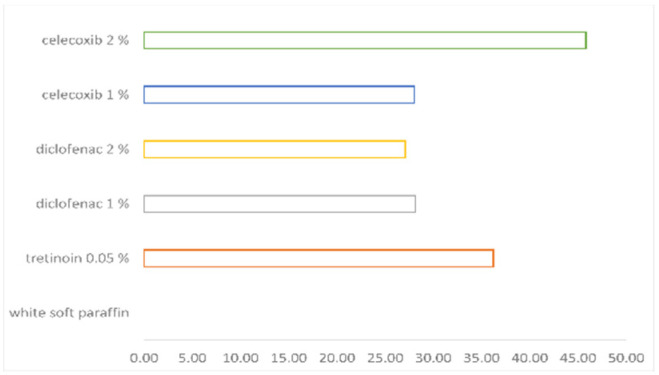
The percentual drug activity.

The induction of the epidermal differentiation, as a marker of orthokeratosis degree, was in the following order: celecoxib 2% > tretinoin 0.05% > diclofenac 1% > celecoxib 1% > diclofenac 2% > white soft paraffin > untreated mice (see Table 1). The celecoxib 2% effect on orthokeratosis degree is superior to tretinoin effect (see Figure 4). The percentual drug activity was the greatest for celecoxib 2% (45.84%) (see Figure 5).

**Figure 6:**
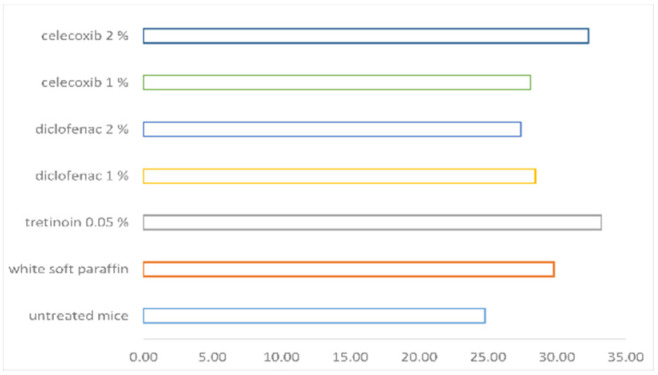
The mean epidermal thickness.

The order regarding the mean epidermal thickness was: tretinoin 0.05% > celecoxib 2% > white soft paraffin > diclofenac 1% > celecoxib 1% > diclofenac 2% > untreated mice (see Table 1; see Figure 6).

There is statistical significance between the orthokeratosis degree obtained in negative control groups (white soft paraffin or untreated mice) and the orthokeratosis degree of the positive control (tretinoin 0.05%) and also the NSAIDs groups (celecoxib 2%, diclofenac 1%, celecoxib 1%, diclofenac 2%) (see Table 2).

The orthokeratosis degree obtained for the celecoxib 2% group is at the limit of statistical significance level (*p* = 0.055), being higher in comparison with the orthokeratosis degree obtained in the other test substance group from the NSAIDs class (celecoxib 1%, diclofenac 2%, diclofenac 1%) (see Table 2).

Regarding mean epidermal thickness, there is a statistical difference between celecoxib 1%, celecoxib 2%, tretinoin 0.05%, white soft paraffin and the untreated mice. The diclofenac 1% and diclofenac 2% were not different in comparison with untreated mice but were significant in comparison with the positive control group (tretinoin 0.05%). Celecoxib 2% and tretinoin 0.05% increased similarly the mean epidermal thickness (see Table 3).

## 4. Discussion

The in vivo lab mouse experiments used to analyze the potential anti-psoriasis effect of the topically applied substances are mainly represented by the imiquimod model and the tail model for psoriasis. Psoriasis is a complex inflammatory and autoimmune disease that does not appear naturally in mice. The existing literature models can evaluate only some aspects of the disease pathogeny [16].

We tested two substances from the NSAIDs class, diclofenac and celecoxib, administered topically, mixed in a lipophilic base ointment, in the in vivo tail model for psoriasis. This model is based on the morphometric analysis of the effect of tested substances on keratinocyte differentiation highlighted by the orthokeratosis degree and by the mean epidermal thickness. Orthokeratosis degree was measured as a percentual ratio of the horizontal continuous granular layer relative to the horizontal length of the whole epidermal scale, between two adjacent hair follicles. The mean epidermal thickness was measured from the dermo-epidermic junction to the inferior part of the stratum corneum [17].

Some NSAIDs can have anti-proliferative properties and hence an anti-psoriasis effect. Recently, in the field of topical treatment for some dermatological diseases characterized by aberrant keratinocyte proliferation (actinic keratosis), some agents from the NSAIDs class were introduced, such as diclofenac and piroxicam [18]. Their mechanism of action consists of blocking COX-2 with a reduction in angiogenesis and cellular proliferation. Recent studies showed that diclofenac is involved in cellular apoptosis regulation as it destroys dysplastic keratinocytes by cellular programmed death [11]. After topical administration, diclofenac can penetrate skin and is absorbed in the deep epidermis where it can reach concentrations that exert an anti-inflammatory therapeutic effect in arthritis. Diclofenac is a potent topical inhibitor of COX-2 with one of the greatest anti-inflammatory indexes for COX-2 inhibition in the dermis [19]. In order to evaluate the effects of NSAIDs on the granular layer, we selected diclofenac, a non-selective COX inhibitor, and celecoxib, a predominant COX-2 inhibitor.

We selected a lipophilic ointment base as vehicle for the tested substances, as it permits an easy application and a good absorption for the active substances.

From the data presented in our study, it can be hypothesized that some NSAIDs with non-selective COX inhibitor activity, such as diclofenac, and a more selective COX-2 inhibitor, such as celecoxib, have an anti-psoriasis effect comparable with tretinoin, in a mouse tail model. Retinoids (tretinoin) are well-known substances with anti-proliferative and anti-psoriasis effects in rodents’ psoriasis models [20,21].

Tretinoin 0.05% in white soft paraffin increased significantly the orthokeratosis degree versus negative controls (untreated and white soft paraffin groups). This fact describes an antiproliferative effect and certifies the validity of our tail model. Literature data show that tretinoin stimulates squamous layer detachment [22]. Retinoids limit hyperproliferation, decrease inflammation and rebuild the normal epidermal differentiation. Their mechanism of action is complex, as they interact with the retinoid acid receptors (RAR, RXR) localized in the cell nucleus, blocking the release of pro-inflammatory mediators [23].

The NSAIDs tested in the present study (diclofenac 1% and 2%, celecoxib 1% and 2%) increased significantly the orthokeratosis degree, based on granular layer development, when compared with both negative controls (see Table 1). Celecoxib 2% had the highest orthokeratosis degree, with a difference at the limit of statistical significance (*p* = 0.055) when compared with celecoxib 1%, diclofenac 1% and diclofenac 2%.

Percentual drug activity is a derived parameter from the orthokeratosis degree parameter, which represents the percentual intensity of the orthokeratosis effect of a given substance in relation to the maximum intensity of this effect. The importance of this parameter is its utility to compare more substances, even in different experimental conditions.

Celecoxib 2% effect on orthokeratosis degree was slightly more intense than the tretinoin effect, the last being a well-known anti-proliferative substance. The greatest percentual drug activity was for celecoxib 2% (45.84%).

Mean epidermal thickness was measured only from the dermo-epidermal junction to the inferior part of the stratum corneum and did not take into consideration the stratum corneum. This parameter was significantly increased in the groups treated with celecoxib 1% and 2% and with tretinoin 0.05%, but it was not significant for the diclofenac groups (1% and 2%) when compared with untreated mice. The group treated with white soft paraffin also had a significant rise in mean epidermal thickness versus untreated mice. Although diclofenac did not increase significantly the mean epidermal thickness, it induced orthokeratosis and had an anti-proliferative effect. On the other hand, white soft paraffin had a comparable effect on mean epidermal thickness as celecoxib 1% and 2% and the positive control, tretinoin 0.05%, versus untreated mice, but did not have an anti-proliferative role, as it did not change the degree of orthokeratosis (see Table 3).

Mean epidermal thickness can only be corroborated with the orthokeratosis degree in order to evaluate the anti-psoriasis effect. The white soft paraffin group increased mean epidermal thickness but not the orthokeratosis degree, so it did not have an anti-psoriasis effect.

Taking into account that celecoxib had the greatest percentual drug activity, inducing the highest level of orthokeratosis degree, even higher than a well-known antiproliferative substance (tretinoin), we searched literature data that could explain the mechanism involved in the action of celecoxib on psoriasis in mice.

COX-1 and COX-2 are enzymes with distinct roles in various physiologic processes. COX-1 is constitutively expressed in multiple cells, while COX-2 is considered an inducible enzyme by multiple and diverse inflammatory stimuli. At therapeutic concentrations, celecoxib inhibits minimally COX-1, having a COX-2 inhibition intensity almost 30 times higher than that of COX-1 [13,14].

The keratinocyte proliferation induced by prostaglandin E-2 (PGE-2) is common in multiple signaling pathways such as epidermal growth factor receptor (EGFR)-proto-oncogene RAS-extracellular signal regulated kinase (ERK) (EGFR-Ras-ERK), phosphoinositide-3-kinase -protein kinase (PK) B (PI3-K-Akt), cAMP-PKA, transcription factors, activating protein-1 (AP-1) and nuclear factor kB (NF-kB) [24,25,26]. From this point of view, it can be stated that COX-2 is a target in different diseases characterized by aberrant keratinocyte proliferation, such as psoriasis. This mechanism might explain the possible involvement of PGE-2 in neoplastic proliferation, by apoptosis inhibition [24,27,28,29].

AP-1 is one of the major eukaryotic transcription factors involved in regulating COX-2 expression. AP-1 is minimally activated under normal physiologic conditions but is dramatically activated by various pathophysiological stimuli. The activity of AP-1 is regulated at the level of transcription of c-jun and c-fos genes. The topical application of celecoxib suppressed the expression of c-Jun and c-Fos protein induced by 12-O-tetradecanoylphorbol-13-acetate (TPA), which can contribute to the antitumor-promoting effect of celecoxib on mouse skin carcinogenesis [13]. Actinic keratosis, psoriasis and cutaneous carcinoma imply aberrant keratinocyte hyperproliferation, so it is possible that an AP-1 mechanism is involved.

Furthermore, celecoxib inhibits sodium current through sodium voltage-dependent channels from the dorsal ganglia of the rat. This fact can contribute to its analgesic effect [30]. Celecoxib also inhibits some types of potassium channels and L-type calcium channels, having an anti-inflammatory effect [31].

Therefore, along with the COX-2 inhibition, celecoxib might have an anti-psoriasis effect by other independent mechanisms.

The limitation of our study consists in the fact that we used only two substances from the NSAIDs class, e.g., diclofenac and celecoxib, so we cannot draw a conclusion regarding the anti-psoriasis effect of the whole NSAIDs class.

## 5. Conclusions

The tail model for psoriasis is a valid model in measuring the anti-psoriasis effect of some topical applied substances.

As orthokeratosis is the most important parameter for the anti-psoriasis effect, all tested NSAIDs class groups (celecoxib 1% and 2% and diclofenac 1% and 2%) increased this parameter versus control groups, and celecoxib 2% tended to have a significantly higher effect than celecoxib 1%, diclofenac 1% or diclofenac 2%groups (*p*=0.055). Percentual drug activity increased in the same way as the orthokeratosis degree and was found to be the greatest for celecoxib 2%.

Although all substances tested, including white soft paraffin, increased the mean epidermal thickness versus untreated mice (statistically significant or non-significant), only diclofenac, celecoxib and tretinoin induced variant orthokeratosis degrees, showing a possible anti-psoriasis effect. In the group treated with white soft paraffin, the orthokeratosis degree remained unchanged.

Along with the COX-2 inhibition, celecoxib might have an anti-psoriasis effect by other independent mechanisms.

The values obtained in the case of celecoxib 2% ointment regarding the orthokeratosis degree and percentual drug activity are providing premises for further investigations regarding this effect and the mechanism of action involved.

The present study brings new data regarding the in vivo anti-psoriasis effect of the NSAIDs drug class, justifying future clinical studies on the use of this drug class for topical psoriasis treatment.

## Figures and Tables

**Table 1:** The effect of tested substances expressed as orthokeratosis degree (as percent), mean epidermal thickness and percentual drug activity.

	ORTHOKERATOSIS DEGREE	MEAN EPIDERMAL THICKNESS	PERCENTUAL DRUG ACTIVITY
UNTREATED MICE	17.7 ± 1.81	24.77 ± 2.43	
WHITE SOFT PARAFFIN	19.64 ± 3.2	29.76 ± 2.02	0
TRETINOIN 0.05%	48.72 ± 6.09	33.22 ± 2.61	36.19
DICLOFENAC 1%	42.24 ± 13.73	28.42 ± 2.62	28.12
DICLOFENAC 2%	41.42 ± 8.17	27.38 ± 2.75	27.10
CELECOXIB 1%	42.15 ± 7.28	28.07 ± 2.37	28.01
CELECOXIB 2%	56.48 ± 11.33	32.28 ± 6.18	45.84

**Table 2:** Multiple statistical comparisons regarding orthokeratosis using Kruskal–Wallis test.

Gropus	Untreated Mice	White Soft Paraffin	Tretinoin 0.05%	Diclofenac 1%	Diclofenac 2%	Celecoxib 1%	Celecoxib 2%
Untreated mice		N (0.361)	S (0.006)	S (0.006)	S (0.006)	S (0.006)	S (0.006)
White soft paraffin	N (0.361)		S (0.004)	S (0.004)	S (0.004)	S (0.004)	S (0.004)
Tretinoin 0.05%	S (0.006)	S (0.004)		N (0.109)	N (0.262)	N (0.337)	N (0.337)
Diclofenac 1%	S (0.006)	S (0.004)	N (0.109)		N (0.837)	N (0.423)	N (0.055)
Diclofenac 2%	S (0.006)	S (0.004)	N (0.262)	N (0.837)		N (1)	N (0.055)
Celecoxib 1%	S (0.006)	S (0.004)	N (0.337)	N (0.423)	N (1)		N (0.055)
Celecoxib 2%	S (0.006)	S (0.004)	N (0.337)	N (0.055)	N (0.055)	N (0.055)	

S = statistically significant; N = statistically non-significant; level of statistical significance is at *p* ≤ 0.05.

**Table 3:** Multiple statistical comparisons regarding mean epidermal thickness using Kruskal–Wallis test.

Gropus	Untreated Mice	White Soft Paraffin	Tretinoin 0.05%	Diclofenac 1%	Diclofenac 2%	Celecoxib 1%	Celecoxib 2%
Untreated mice		S (0.018)	S (0.006)	N (0.1)	N (0.201)	S (0.045)	S (0.028)
White soft paraffin	S (0.018)		N (0.078)	N (0.423)	N (0.055)	N (0.262)	N (1)
Tretinoin 0.05%	S (0.006)	N (0.078)		S (0.037)	S (0.016)	S (0.025)	N (0.337)
Diclofenac 1%	N (0.1)	N (0.423)	S (0.037)		N (0.631)	N (0.749)	N (0.337)
Diclofenac 2%	N (0.201)	N (0.055)	S (0.016)	N (0.631)		N (0.631)	N (0.078)
Celecoxib 1%	S (0.045)	N (0.262)	S (0.025)	N (0.749)	N (0.631)		N (0.262)
Celecoxib 2%	S (0.028)	N (1)	N (0.337)	N (0.337)	N (0.078)	N (0.262)	

S = statistically significant; N = statistically non-significant; level of statistical significance is at *p* ≤ 0.05.

## Data Availability

Data will be available upon request.
